# Dedifferentiated fat cells-derived exosomes (DFATs-Exos) loaded in GelMA accelerated diabetic wound healing through Wnt/β-catenin pathway

**DOI:** 10.1186/s13287-025-04205-9

**Published:** 2025-02-28

**Authors:** Miao Dong, Xuan Ma, Facheng Li

**Affiliations:** Department of Body Contouring and Fat grafting Center, Plastic Surgery Hospital, Chinese Academy of Medical Sciences and Peking Union Medical College, No. 33 Badachu Road, Shijingshan District, Beijing, 100144 People’s Republic of China

**Keywords:** Diabetic wound healing, Dedifferentiated fat cells, Exosomes, GelMA hydrogel, Wnt / β-catenin pathway

## Abstract

**Background:**

Diabetic foot ulcers pose significant challenges for clinicians worldwide. Cell-free exosome therapy holds great potential for wound healing. Dedifferentiated fat cells (DFATs) have been used in tissue engineering and regeneration, but there are no reports on the use of DFATs-derived exosomes in diabetic wound repair.

**Objectives:**

This study aims to investigate whether DFATs-Exos accelerated diabetic wound healing and explore its potential mechanism.

**Methods:**

In vitro, DFATs-Exos were harvested from adipose tissue and used to treat endothelial cells (ECs) and fibroblasts. XAV939 was used as a Wnt/β-catenin pathway inhibitor. The biocompatibility of gelatin methacryloyl (GelMA) hydrogel was assessed. In vivo, DFAT-derived exosomes were encapsulated in 10% GelMA hydrogel and applied to a diabetic wound model. Histological analysis and wound closure rates were evaluated.

**Results:**

DFATs-Exos promoted angiogenesis in ECs and significantly alleviated the high glucose-induced inhibition of cell proliferation and migration by activating the Wnt/β-catenin pathway. In vivo, compared to DFAT-Exos or GelMA alone, the DFAT-Exos/GelMA combination accelerated wound closure and enhanced collagen maturity.

**Conclusion:**

The DFAT-Exos/GelMA hydrogel significantly promoted wound healing in a diabetic animal model through activation of the Wnt/β-catenin signaling pathway.

**Supplementary Information:**

The online version contains supplementary material available at 10.1186/s13287-025-04205-9.

## Introduction

Diabetes is a prevalent chronic disease worldwide, and diabetic foot ulcer (DFU) is a serious complication resulting from long-term poor glycemic control [1]. Among approximately 537 million diabetes patients globally, 19–34% develop DFU. Of those who develop DFU, 20% ultimately require amputation, and 10% die within one year [2, 3]. DFU remains a major challenge in global healthcare. Therefore, developing effective methods for diabetic wound healing is a crucial focus of current medical research [4].

Exosome therapy for diabetic wounds has shown potential therapeutic benefits [5]. Exosomes can be derived from various sources, including endothelial cells, bone marrow mesenchymal stem cells (BMSCs), macrophages, and adipose-derived stem cells (ADSCs) [6]. However, obtaining enough exosomes requires time-consuming cell culture and expansion [7]. Dedifferentiated fat cells (DFATs) are a type of stem cell derived from mature adipocytes, which can be harvested from discarded adipose tissue after liposuction. Studies have shown that DFATs have promising potential in tissue engineering and regeneration [8, 9]. However, there are no reports on the application of DFAT-derived exosomes in diabetic wound repair. Local application of exosomes has limitations, such as rapid clearance and the need for repeated administration [10]. Encapsulating exosomes in biomaterials can extend their retention time at the wound site without impairing their biological activity. Hydrogels, due to their plasticity and water retention capacity, are considered promising carriers for wound treatment [11]. Gelatin methacryloyl (GelMA) hydrogel, a biomaterial with low immunogenicity, good biocompatibility, and favorable physicochemical properties, has garnered increasing attention. GelMA hydrogels with appropriate pore sizes provide a stable scaffold for angiogenesis and fibroblast migration [12].

In this study, we isolated DFATs and extracted exosomes for the first time. By exposing human dermal fibroblasts (HDFs) to a high-glucose (HG) environment, we established an in vitro diabetic model and investigated the wound healing potential of DFAT-Exos. Our results demonstrated that DFAT-Exos significantly mitigated HG-induced inhibition of cell proliferation and migration by activating the Wnt/β-catenin pathway.

## Method

### Cell isolation and culture

This study was approved by the Ethics Committee of Plastic Surgery Hospital, Chinese Academy of Medical Sciences (2024 (334)) and was conducted in accordance with the principles of the Declaration of Helsinki. An inform consent had been signed with patients and the relatives before the usage of their tissue samples. Adipose tissue was obtained from three patients who underwent liposuction surgery, and skin was obtained from two patients who underwent abdominoplasty.

DFATs were obtained using the ceiling culture method as previously described [13]. Briefly, 2 ml of purified adipocytes were put into a T25 flask (Corning, USA) full of Dulbecco’s modified Eagle’s medium (DMEM, Invitrogen, USA) supplemented with 20% fetal bovine serum (FBS, Gibco, USA), and were incubated at 37℃ in 5% CO_2_. The flask was inverted to allow adipocytes to distribute evenly on the ceiling. After 14 days of culturing, the adipocytes gradually lost large lipid, became spindle-like cells, and proliferate to reach confluence. The adherent DFATs were used to extract exosomes. A human MSC analysis kit (BD, USA) was used to identify DFATs.

Human dermal fibroblasts were extracted through tissue adhesion method. Human dermal tissue was cut into 1 × 1 mm pieces and placed in a 10 cm culture dish (Corning, USA). The culture condition was DMEM supplemented with 10% FBS and penicillin/streptomycin. (Gibco, USA) The culture medium was changed slightly every 3–4 days. After three weeks, fibroblasts migrated out from the tissue. Passage 3 fibroblasts were used for further research.

### Exosomes isolation and characterization

Exosomes were isolated from the supernatant using ultracentrifugation method [14]. After starving for 48 h, the DFATs supernatant was collected in ultracentrifugation tubes (HITACHI, Japan). Exosomes were collected by centrifugation at 100,000 g for 70 min at 4℃. Exosomes were characterized using transmission electron microscopy (TEM), nanoparticle tracking analysis (NTA), and Western blot. The following primary antibodies were used: anti-CD9(BM4212, 1:1500; Boster, China), CD63(BS-43117R; 1:2000; Bioss, China), TSG101(BM4281, 1:1500; Boster, China), Calnexin (ab92573; 1:4000; Abcam, USA). Final exosomes were obtained and stored at − 80 °C for further study.

### Preparation and characterization of GelMA hydrogel

GelMA hydrogel was prepared by dissolving 0.6 g GelMA powder in PBS with a magnetic stirrer at 37 °C for 1 h (EFL Co., China). The solution was sterilized using a 0.22 μm filter for future usage (Millipore, USA). GelMA hydrogels at concentrations of 5%, 10%, and 15% were prepared.

#### SEM analysis of GelMA hydrogel

Hydrogels of different concentrations were fixed in 2.5% glutaraldehyde solution (Solarbio, China) overnight at 4 °C, dehydrated with a gradient ethanol series, and observed under a scanning electron microscope (Tescan Clara, Czech) to capture high-resolution images of the microstructure.

#### Hydrogel degradation and release assay

Cylindrical samples of GelMA hydrogels containing 200 µg/mL DFATs exosomes were prepared and crosslinked under 405 nm blue light (diameter 10 mm, height 5 mm). The crosslinked hydrogels were immersed in PBS in a 12-well plate and incubated at 37 °C. Samples were weighed daily, and the supernatant was collected every 24 h to measure the release rate using the BCA assay (Beyotime, China).

$$\displaylines{ Degration\,rate = \cr {{current\,weight} \mathord{\left/{\vphantom {{current\,weight} {\operatorname{int} ial\,weight \times 100}}} \right.\kern-\nulldelimiterspace} {\operatorname{int} ial\,weight \times 100}}\% \cr} $$

$$\displaylines{\operatorname{Re} lease\,rate = \cr {{current\,protein} \mathord{\left/{\vphantom {{current\,protein} {total\,protein \times 100}}} \right.\kern-\nulldelimiterspace} {total\,protein \times 100}}\% \cr} $$

#### Viability of fibroblasts co-cultured with GelMA

50 ul/well GelMA solution was injected into 96-well plate and irradiated with 405 nm light to crosslink. Fibroblasts were inoculated and incubated in an incubator at 37° C and 5% CO_2_. At 1d, 3d, 5d and 7d, the effects of GelMA on cell proliferation were assessed using the CCK-8 method. (Beyotime, China)

### In vitro test

#### DFATs-Exos internalization

DFATs-Exos were labeled with PKH26 Cell Linker Kit (Sigma-Aldrich, USA). Labeled exosomes or exsomes loaded in GelMA were co-cultured with fibroblasts in FBS-free DMEM (Gibco, USA) for 6 h and 24 h. After co-staining with 4,6-diamidino-2-phenylindole (DAPI, Sigma, USA), fibroblasts were observed under a confocal microscope (Leica, Germany).

#### Angiogenesis assay

The angiogenic potential of exosomes was evaluated using a tube formation assay and ELISA. Human umbilical vein endothelial cells (HUVECs) were purchased from Wuhan Procell Corporation. Matrigel (Corning, USA) was added to a 96-well plate (100 µl per well), and HUVECs (2 × 10^4^ cells/well) were seeded onto the Matrigel and treated with either DMEM or DMEM supplemented with exosomes (50 µg/ml). After 9 h of incubation, the results were analyzed using ImageJ software.

Supernatants from treated endothelial cells were collected, and VEGF and HGF concentrations were measured using an ELISA kit (Jianglai biology, Shanghai, China) according to the manufacturer’s instructions. Absorbance at 450 nm was determined using a microplate reader.

#### Proliferation and migration assays

Fibroblast proliferation was assessed using an EdU cell proliferation kit (Beyotime, China) according to the manufacturer’s instructions. After 6–8 h of serum starvation in fibroblasts, a series concentration of glucose (0mM, 5.5mM, 25mM, 35mM) added in DMEM was used to testify the viability of fibroblasts through CCK-8 assay. High-glucose (HG) DMEM was used to mimic the diabetic hyperglycemic environment. XAV939(Selleck, China) was a small molecule inhibitor that selectively blocks Wnt /β-catenin-mediated transcription by inhibiting tankyrase 1 and tankyrase 2 [15].

35mM HG-DMEM was used to mimic the diabetic cell model. The experimental groups were divided as follows: (a) Control: Fibroblasts cultured in DMEM. (b) HG: Fibroblasts cultured in 35mM high-glucose DMEM. (c) HG + Exos: Fibroblasts cultured in 35mM high-glucose DMEM with 50 µg/ml exosomes. (d) HG + XAV939 + Exos: Fibroblasts cultured in 35mM high-glucose DMEM with 50 µg/ml exosomes and 10 µM XAV939. After 4 h of EdU staining, images were captured using a fluorescence microscope (Leica, Germany).

Fibroblast migration was evaluated using a Transwell assay (Corning, USA). Fibroblasts (1 × 10⁴ cells/well) were seeded into the upper compartment of the Transwell, and 20% FBS DMEM was added to the lower compartment. The groups were the same as those used in the EdU test. After 24 h, cells that migrated to the lower surface were stained with 0.1% crystal violet (Solarbio, Beijing, China) and counted under a microscope.

#### Western blot of fibroblasts

Firstly, the efficacy of XAV939 was verified through Western blot. 10 µM XAV939 was added to the fibroblast culture medium, and after 48 h, proteins were extracted to assess the expression of Wnt and β-catenin. Relative protein expression was calculated as the ratio of target protein to GAPDH.

Secondly, after culturing under HG, HG + Exos, or HG + Exos + XAV939 conditions, total protein was extracted from fibroblasts using RIPA lysis buffer containing protease inhibitors (MedChemExpress, China). Protein concentrations were determined using a BCA kit (Beyotime Biotechnology, China). Proteins were separated by SDS-PAGE and transferred onto PVDF membranes (Millipore, USA). Membranes were blocked with 5% BSA and incubated with primary antibodies against α-SMA (1:6000; Proteintech, China), Collagen type 1 (1:2000; Proteintech, China), β-catenin (1:2500; Bioss, China), Wnt (1:2000; Boster, China) and β-actin (1:2000; Boster, China) at 4 °C overnight. After washing, membranes were incubated with HRP-conjugated IgG antibodies (1:10000; Jackson, USA) and developed using ECL detection reagent (Millipore, USA). Bands were visualized using the Bio-Rad system (BioRad, Hercules, CA, USA) and analyzed with ImageJ software. Relative protein expression was calculated as the ratio of target protein to β-actin.

### Diabetic wound healing

#### Animal study design

All animals were housed in the Specific Pathogen Free (SPF) facility and acclimated under standard laboratory conditions and had free access to standard water and food. All procedures were conducted in accordance with the “Guiding Principles in the Care and Use of Animals” (China) and were approved by the Laboratory Animal Ethics Committee of Plastic Surgery Hospital, Chinese Academy of Medical Sciences (2024 animal (152)). The work has been reported in line with the ARRIVE guidelines 2.0.

A total of 32 male BALB/c mice (6–8 weeks old, 17–23 g) were purchased from Vital River Laboratory Animal Technology (Beijing, China). (3 mice/cage) Diabetes was induced by intraperitoneal injection of streptozotocin (STZ, 150 mg/kg) for 7 consecutive days, and blood glucose levels were monitored for 1 week. Mice with blood glucose levels ≥ 16.7 mmol/L were considered diabetic and maintained for 4 weeks. Ten days post-STZ injection, the weight of diabetic mice was significantly lower (21.6 ± 1.8 g) compared to normal mice (24.3 ± 0.6 g). The anesthesia method involved using an inhalation anesthesia machine for isoflurane gas anesthesia. (RWD life technology Co., ShenZhen, China) Oxygen flow was set at 1 L/min, with an induction anesthesia concentration of 3–5% and a maintenance anesthesia concentration of 0.6–1.2%. Under isoflurane anesthesia, hair on the back of diabetic mice was shaved and disinfected. An 8 mm circular mark was created using a skin punch, and full-thickness skin was excised. To minimize skin contraction on wound healing, a silicone ring with a 1 cm inner diameter was fixed around the wound using bio-glue [16]. After different treatments application, the wounds were covered with 3 M semipermeable membrane. The dressings were changed every 2–3 days to prevent contamination. Each mouse was fed individually in separate cages to avoid interference with the wound site. The animal caregivers and investigators were blinded to which intervention each animal received during the experiment.

The mice were randomly divided into four groups: (a) control group (100 µl PBS), (b) GelMA group (100 µl 10% GelMA), (c) Exosomes group (100 µl of 200 µg/ml DFATs-Exos), and (d) DFATs-Exos/GelMA group (100 µl of 200 µg/ml exosomes incorporated in 10%GelMA) (*n* = 8). Wound healing was photographed on days 0, 4, 7, 10, and 14. On days 7 and 14, four mice in each group were randomly chosen to euthanize using CO_2_ and the samples were used for histological staining and Western blot analysis. The animal caregivers and outcome assessors were blinded to which intervention each animal received during the experiment.

#### Histologic analysis

On day 14, re-epithelialization and collagen deposition were assessed using H&E and Masson staining (Solarbio, China). Immunohistochemical staining for CD31 (1:2000; Abcam, USA) and α-SMA (1:1000; Abcam, USA) was performed to evaluate angiogenesis and fibrosis in the wound area. On day 7, immunofluorescence staining for Ki-67 (1 µg/ml; Abcam, USA) and iNOS/CD206 (1:2000; Abcam, USA) was used to analyze cell proliferation and inflammatory responses. Additionally, β-catenin (1:500; Abcam, USA) staining was conducted to investigate the activation of the Wnt/β-catenin pathway.

#### Western blot analysis of wound tissue proteins

Wound skin samples were preserved in liquid nitrogen for future use. Approximately 50 mg of skin was minced and grinded with RIPA lysis buffer on ice. After lysis, the samples were centrifuged at 10,000 g for 15 min and the supernatant was collected. Protein concentrations were determined using a BCA kit (Beyotime Biotechnology, China). Proteins were separated by SDS-PAGE and transferred onto PVDF membranes (Millipore, USA). Membranes were blocked with 5% BSA and incubated with primary antibodies against β-catenin (1:15000; Proteintech, China), Wnt (1:1500; Proteintech, China), CD206 (1:1000; Proteintech, China) and GAPDH (1:5000; Proteintech, China) at 4 °C overnight. Relative protein expression was calculated as the ratio of target protein to GAPDH.

6. Statistical Analysis.

All data were presented as mean ± standard deviation (SD). Statistical analysis was performed using GraphPad Prism 10.1.0 software. Differences between groups were analyzed by t-test or one-way ANOVA. *P*-value < 0.05 was considered statistically significant.

## Results

**Fig. 1:**
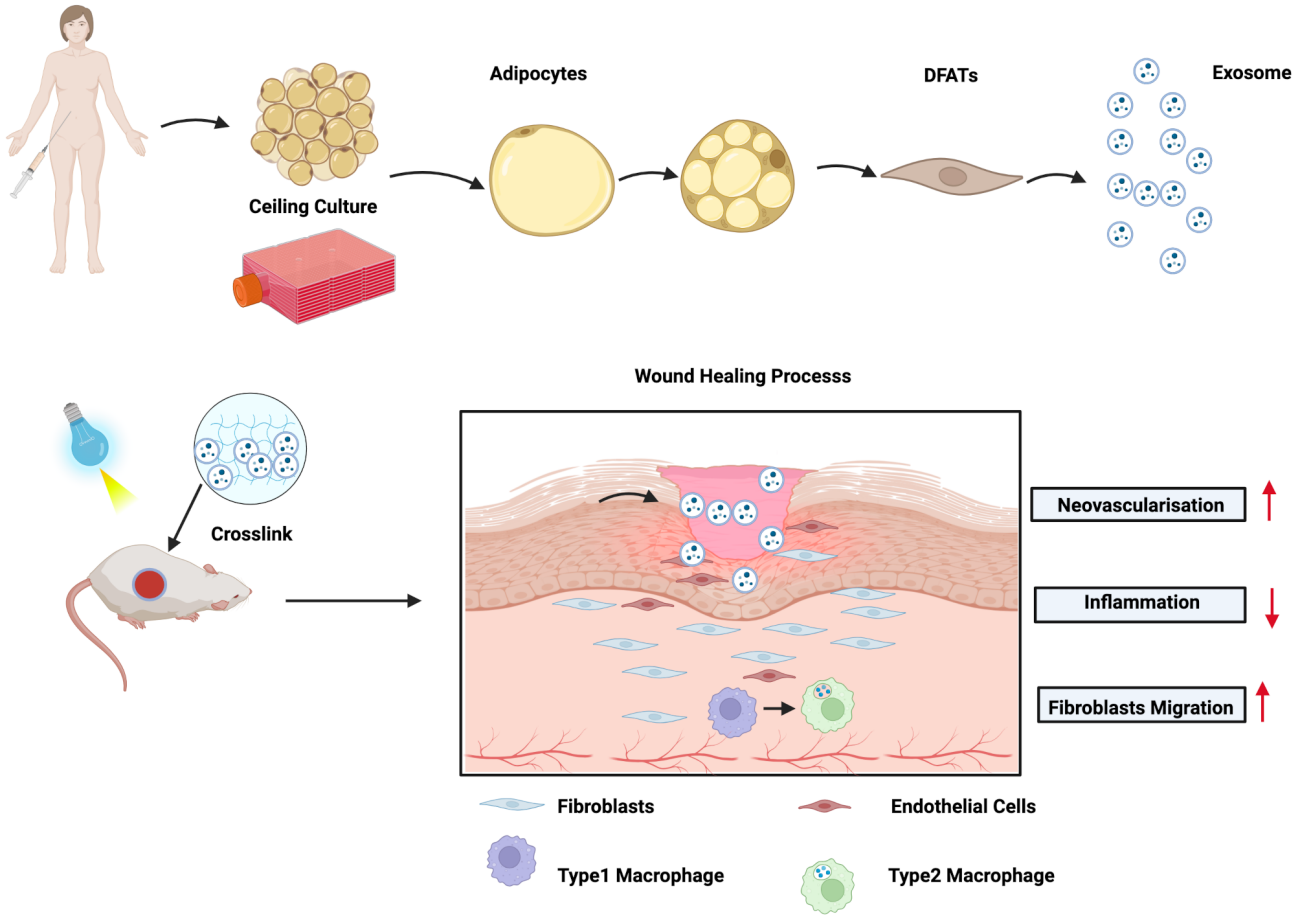
Shows the graphical abstract of this study

The schematic illustration is presented in Fig. 1. (Created with BioRender.com)

### Characterization of DFATs and EVs

**Fig. 2:**
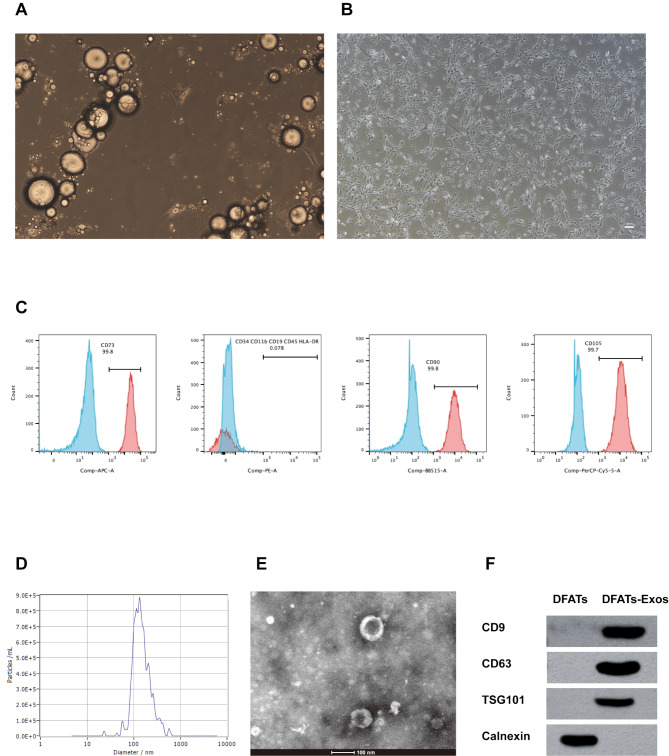
Illustrates the characterization of DFATs and exosomes. DFATs exhibited a spindle-shaped morphology (**A-B**). Flow cytometry analysis showed that DFATs were positive for markers CD90, CD105, and CD73, and negative for CD34, CD11b, CD19, CD45, and HLA-DR (**C**). NTA showed that the average diameter of the exosomes was 136.3 nm (**D**). TEM showed that DFATs-Exos exhibited a typical bilayer membrane structure (**E**). Western blot analysis revealed that DFATs exosomes were enriched in markers such as CD63, CD9, and TSG101, while lacking the marker Calnexin (**F**). Full-length blots are presented in Supplementary Digital Material **1**

DFATs exhibited a spindle-shaped morphology, consistent with previous reports on stem cell characteristics (Fig. 2A and B). Flow cytometry analysis showed that DFATs were positive for markers CD90, CD105, and CD73, and negative for CD34, CD11b, CD19, CD45, and HLA-DR, indicating that high-purity DFATs were successfully obtained (Fig. 2C).

Exosomes were isolated from DFAT culture supernatants. NTA further confirmed that the average diameter of the exosomes was 136.3 nm (Fig. 2D). TEM showed that DFATs-Exos exhibited a typical bilayer membrane structure (Fig. 2E). Additionally, Western blot analysis revealed that DFATs exosomes were enriched in markers such as CD63, CD9, and TSG101, while lacking the marker Calnexin (Fig. 2F), indicating successful isolation of DFATs-derived exosomes. Full-length blots are presented in Supplementary Digital Material 1.

**Fig. 3:**
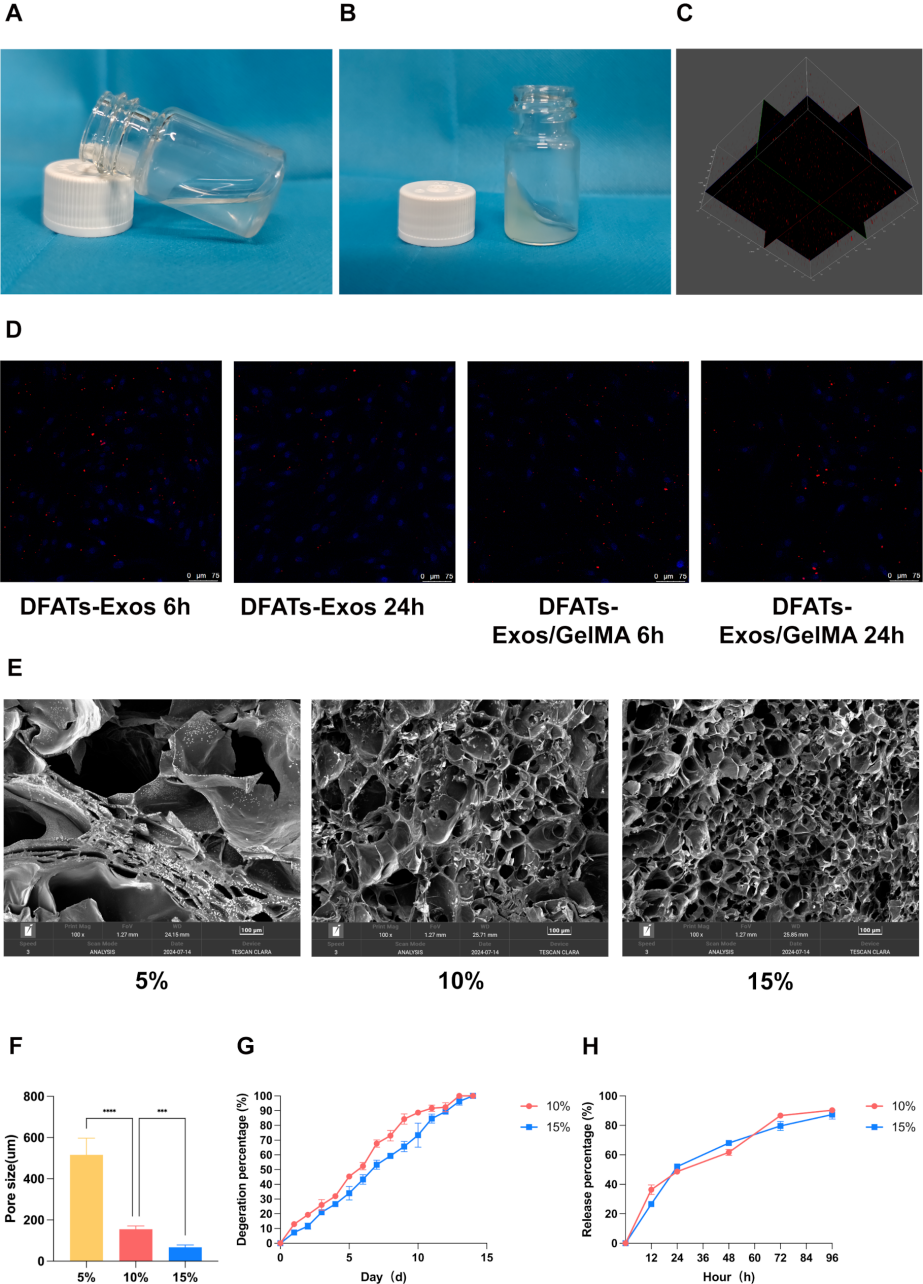
Illustrated the characterization of GelMA. The sterile GelMA solution was in a liquid state (**A**) and formed a hydrogel after crosslinking (**B**). PKH26+ DFATs-Exos were evenly distributed within the GelMA (**C**). After co-culturing with fibroblasts, more PKH26+ exosomes were released and taken up in the Exos/GelMA group (**D**). (**E-F**) showed the microstructure of GelMA. (**G** and **H**) showed the degradation and release rate of GelMA

The sterile GelMA solution was in a liquid state (Fig. 3A), and after crosslinking with 405 nm blue light, it formed a hydrogel (Fig. 3B). To verify the internalization of DFATs exosomes by fibroblasts, exosomes were labeled with PKH26 dye. PKH26 + DFATs-Exos were evenly distributed within the GelMA hydrogel (Fig. 3C). After co-culturing with fibroblasts for 6 h, the red fluorescence signal was localized in the cytoplasm, indicating more release and uptake of DFATs exosomes in Exos group. After 24 h of co-culture, more PKH26 + exosomes were released and taken up in the Exos/GelMA group (Fig. 3D).

### Characterization of GelMA hydrogel

SEM analysis showed that the 5% GelMA hydrogel failed to form a stable porous structure, which may impair oxygen exchange and neovascularization. In contrast, 10% and 15% GelMA hydrogels formed uniform 3D networks with evenly distributed and interconnected pores. The average pore sizes were 155.47 ± 15.56 μm for 10% GelMA and 67.39 ± 11.64 μm for 15% GelMA (Fig. 3E F). Therefore, 10% and 15% GelMA were compared in degradation and release experiments.

Both 10% and 15% GelMA hydrogels were completely degraded within 14 days. On day 4, 10% GelMA degraded by 31.67 ± 1.57%, while 15% GelMA degraded by 26.67 ± 1.53%, indicating that the 10% hydrogel had a faster degradation rate, which is beneficial for wound healing applications (Fig. 3G). In the exosome release experiment, 10% GelMA hydrogels released 61.42 ± 2.08% of exosomes within 48 h, while 15% GelMA released 68.14 ± 4.50%. By day 4, the total protein released by 10% GelMA hydrogels was 90.33 ± 3.86%, compared to 87.09 ± 5.15% by 15% GelMA, with no statistical difference (*P* > 0.05) (Fig. 3H). The 10% GelMA hydrogel provided a more rapid degradation rate, which is advantageous for promoting wound healing. Considering the degradation rate, release rate, and SEM results, 10% GelMA was selected as the optimal concentration for subsequent experiments. The 10% GelMA solution did not exhibit cytotoxic effects on fibroblasts. CCK-8 results of fibroblasts co-cultured with 10% GelMA solution were shown in Supplemental Digital Material 2.

### DFATs-Exos enhance tube formation and VEGF and HGF secretion in HUVECs

**Fig. 4:**
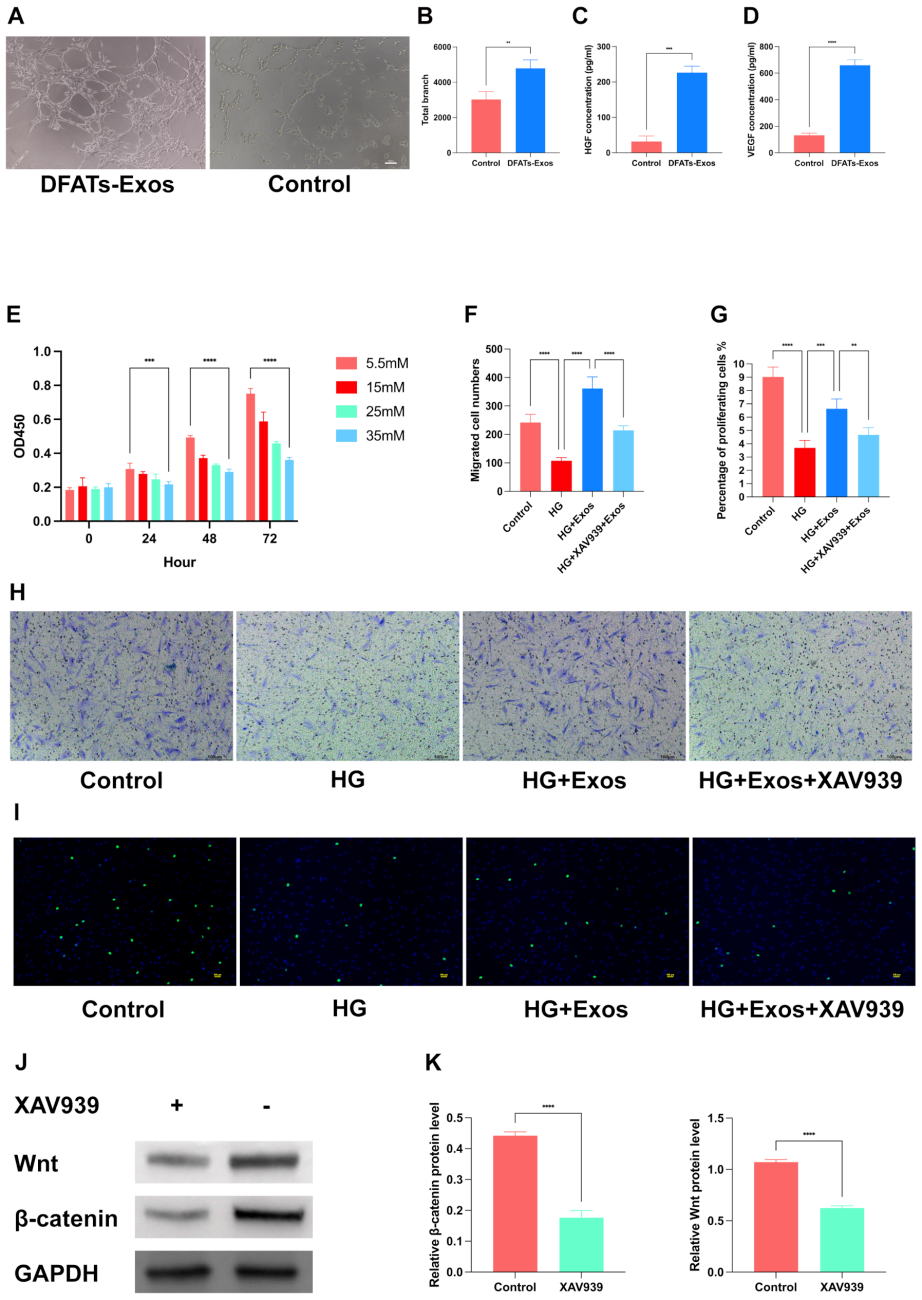
Showed the functions of DFAT-derived exosomes. DFAT-Exos-treated group exhibited significantly more tube formation compared to the control group (*P* < 0.01) (**A-B**). DFAT-Exos group showed significantly higher levels of VEGF and HGF compared to the control group (*P* < 0.001) (**C-D**). 35mM glucose concentration inhibited fibroblasts proliferation significantly and was used for further experiments. (**E**) XAV939 was effective to inhibit the activation of Wnt/β-catenin pathway.(**J-K**) DFATs exosomes promote fibroblast proliferation and migration (**F-I**)

To evaluate the angiogenic potential of DFAT-derived exosomes, a tube formation assay was conducted. After 8 h, the DFAT-Exos-treated group exhibited significantly more tube formation compared to the control group (*P* < 0.01) (Fig. 4A and B). ELISA was performed to measure VEGF and HGF levels in the cell culture supernatant. The DFAT-Exos group showed significantly higher levels of VEGF and HGF compared to the control group (*P* < 0.001) (Fig. 4C and D).

### DFATs-Exos promote fibroblast proliferation and migration in a high-glucose environment

To evaluate the effect of DFATs exosomes on fibroblast proliferation and migration, EdU and transwell assays were conducted. As shown in Fig. 4E, the proliferation of fibroblasts was most significantly inhibited when 35 mM glucose was added to the culture medium. XAV939 effectively inhibited the Wnt /β-catenin pathway. (Figure 4J K) In Fig. 4H, transwell migration assays revealed that the number of migrated cells was significantly higher in the HG + Exos group (107.75 ± 10.01 cells/field) compared to the HG group (361.25 ± 41.34 cells/field), indicating that DFAT-derived exosomes promote fibroblast migration (*P* < 0.0001).

EdU assays demonstrated an increase in the percentage of proliferative cells in the HG + Exos group (6.63 ± 1.82%) compared to the HG group (3.70 ± 0.56%), indicating enhanced cell proliferation due to the influence of exosomes (*P* < 0.001). The addition of a Wnt/β-catenin pathway inhibitor antagonized the therapeutic effects of the exosomes (*P* < 0.01) (Fig. 4I). Inhibition of the Wnt /β-catenin pathway suppressed the protective effect of DFATs-Exos on fibroblasts impaired by high glucose (*P* < 0.001) (Fig. 4F and G).

### DFATs-Exos increased α-SMA, Col 1 and Wnt/β-catenin-related proteins expression in fibroblasts

**Fig. 5:**
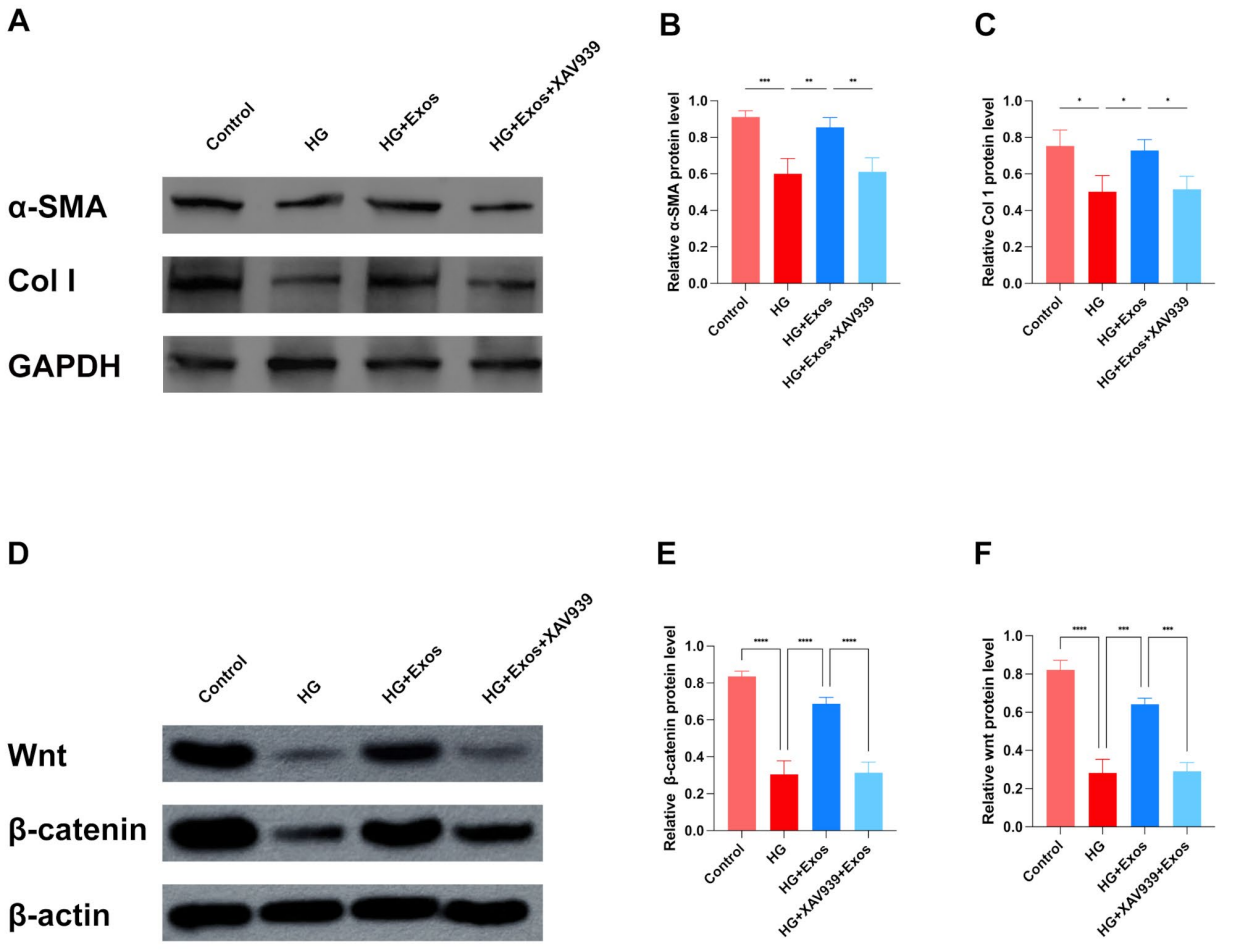
DFATs-Exos increased Wnt/β-catenin-related proteins expression in fibroblasts. (**A**) showed that the expression of α-SMA and Col 1 was markedly decreased in the HG environment. However, upon the addition of DFATs-Exos, α-SMA and Col 1 expression was elevated. The expression of Wnt and β-catenin was markedly decreased in the HG environment. After adding DFAT-Exos, Wnt/β-catenin signaling was reactivated. Moreover, XAV939, an inhibitor of the Wnt/β-catenin pathway, significantly impaired the function of DFAT-Exos. As expected, compared to the control group, XAV939 significantly blocked the activation of the Wnt/β-catenin signaling pathway. (*P* < 0.001) (**D-F**)

Consistent with the migration assay, high-glucose stimulation reduced α-SMA and Col 1 expression in fibroblasts. The addition of DFAT-derived exosomes increased α-SMA and Col 1 expression, while the addition of a Wnt pathway inhibitor XAV939 decreased α-SMA and Col 1 expression. (Figure 5A C) Full-length blots are presented in Supplemental Digital Material 3.

The Wnt/β-catenin signaling pathway is known to be closely associated with wound healing [17]. This study explored whether Wnt/β-catenin signaling is involved in the wound healing effects of DFAT-derived exosomes. Western blot analysis showed that the expression of Wnt and β-catenin was markedly decreased in the HG environment. However, upon the addition of DFAT-Exos, Wnt/β-catenin signaling was reactivated. Moreover, XAV939, an inhibitor of the Wnt/β-catenin pathway, significantly impaired the function of DFAT-Exos. As expected, compared to the control group, XAV939 significantly blocked the activation of the Wnt/β-catenin signaling pathway. (*P* < 0.001) (Fig. 5D F) Full-length blots are presented in Supplemental Digital Material 1 and 4.

### DFATs exosomes promoted wound healing

**Fig. 6:**
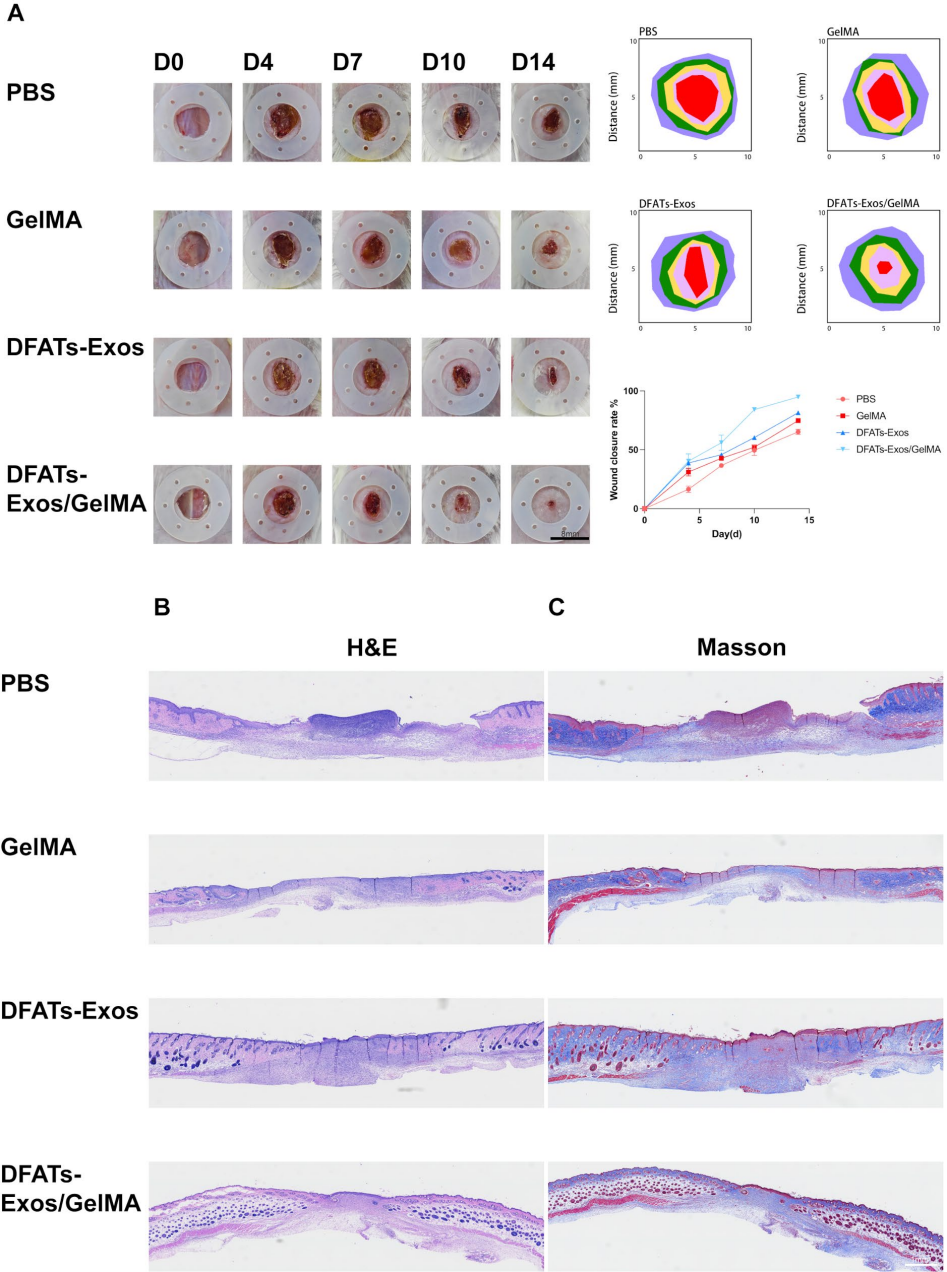
Showed that DFATs-Exos promoted wound healing. DFATs-Exos/GelMA group exhibited the most significant improvement (**A**). (H&E and Masson’s staining were used to evaluate re-epithelialization and collagen remodeling (**B**, **C**)

GelMA hydrogel containing DFATs exosomes demonstrated effective angiogenesis and fibroblast recruitment *in vitro.* To further investigate the wound healing effects of the exosome-loaded hydrogel, we utilized a diabetic animal model. Mice were randomly divided into four groups: PBS group, GelMA group, DFATs-Exos group, and DFATs-Exos/GelMA group. Photographs were taken on day 0, 4, 7, 10, and 14. At each time point, wound healing in all experimental groups was faster than in the PBS group, with the exosome + hydrogel group exhibiting the most significant improvement (Fig. 6A).

H&E and Masson’s staining were used to evaluate re-epithelialization and collagen remodeling. On day 14, the PBS group showed incomplete re-epithelialization, while the remaining groups exhibited good re-epithelialization. DFATs-Exos/GelMA group and the DFATs-Exos group had more granulation tissue and skin appendages regeneration (Fig. 6B). Masson’s staining showed greater collagen deposition in the DFATs-Exos/GelMA group (Fig. 6C).

**Fig. 7:**
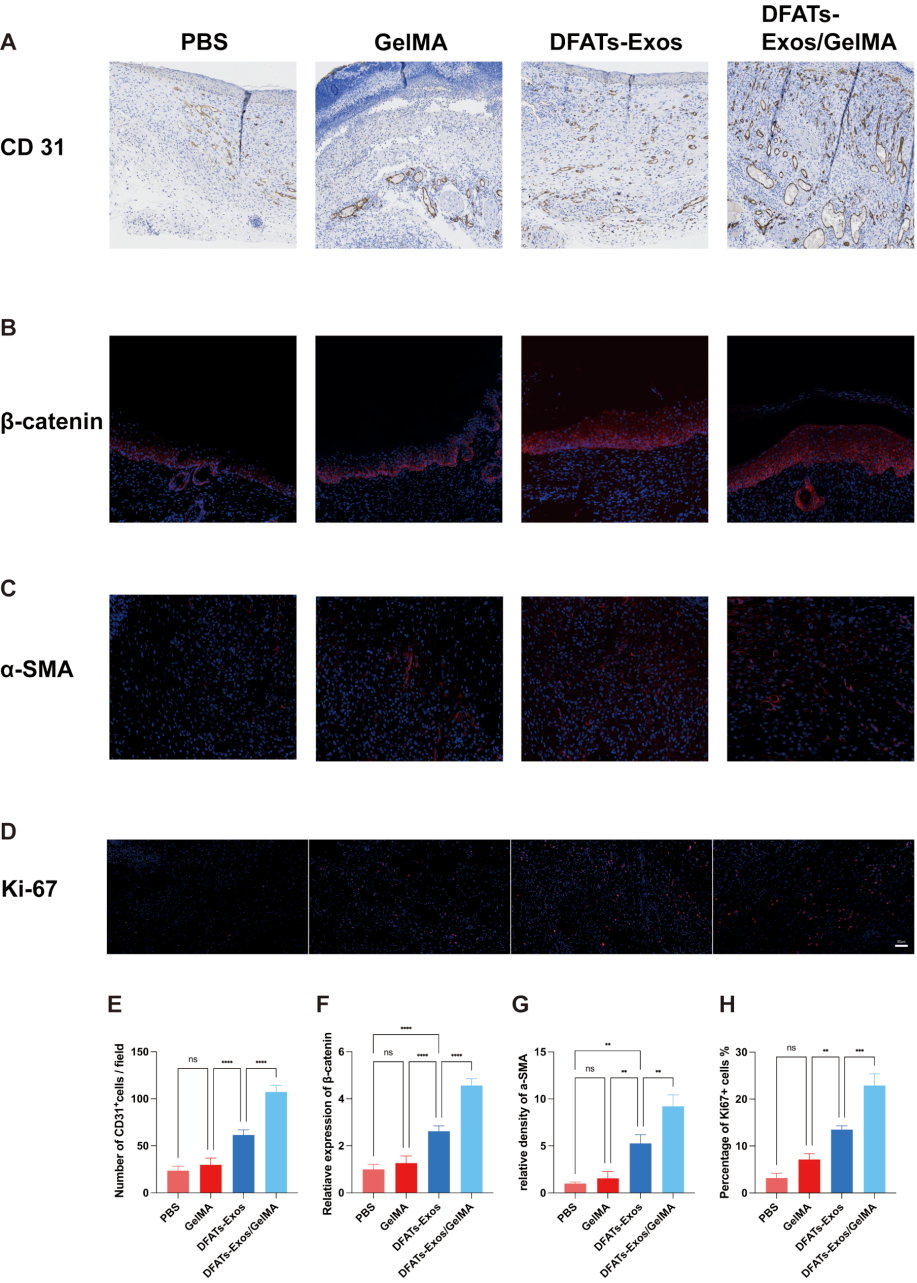
Showed the staining of CD31 (**A**), β-catenin (**B**), α-SMA (**C**), and Ki67 (**D**). DFATs exosomes loaded in GelMA hydrogel promotes improved tissue regeneration, characterized by increased collagen deposition, enhanced angiogenesis and cell proliferation

CD31 staining demonstrated the highest number of mature blood vessels in the DFATs-Exos/GelMA group (107.25 ± 6.85/field) compared to PBS group (23.50 ± 4.79/field), GelMA group (29.75 ± 7.14/field) and DFATs-Exos group (61.50 ± 5.45/field), indicating enhanced angiogenesis on day 14(*P* < 0.0001) (Fig. 7A). The relative expression of β-catenin was also highest in DFATs-Exos/GelMA group, indicating the downstream activation of Wnt/β-catenin pathway (Fig. 7B).

α-SMA, a typical fibrosis marker, played a critical role in the host’s defense response to infection and injury. After injury, fibroblasts were activated and transformed into myofibroblasts, which express α-SMA, promoting granulation tissue formation and wound healing in the damaged area. DFATs exosomes enhanced the transformation of fibroblasts into myofibroblasts, accelerating the healing of diabetic wounds. (Fig. 7C).

**Fig. 8:**
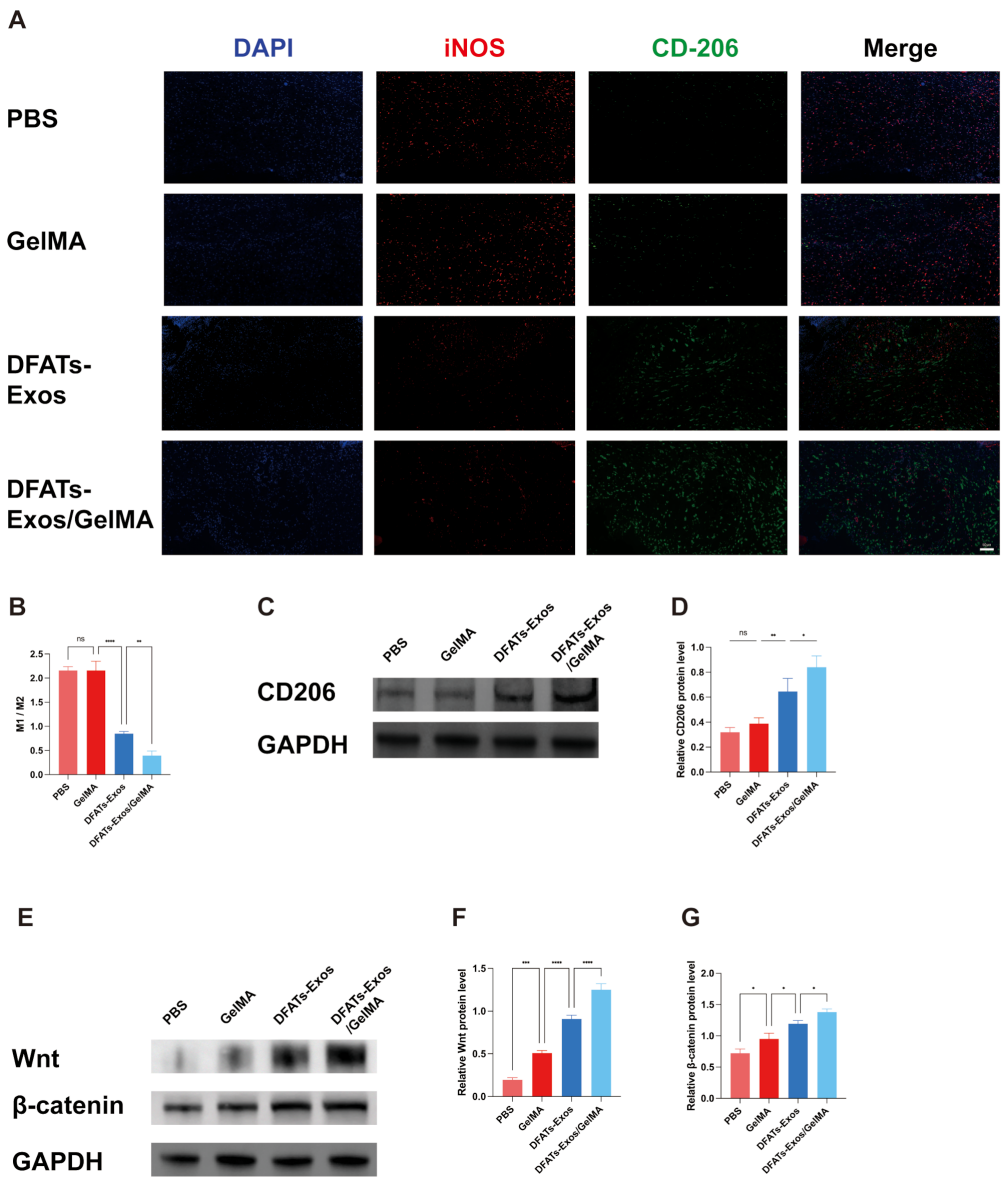
Showed the staining of iNOS, CD206 and western blot of skin wound tissue. The DFATs-Exos/GelMA group exhibited fewer iNOS+ M1 macrophages and more CD206+ M2 macrophages, suggesting a phenotype shift from M1 to M2 (**A**, **B**). Western blot significantly revealed activation of Wnt/β-catenin signaling when DFATs-Exos were added. (*P* < 0.05) Using GelMA hydrogel for the sustained release of DFATs-Exos, the DFATs-Exos/GelMA group exhibited faster wound healing with maximal Wnt/β-catenin pathway activation. (**E-G**)

To evaluate cell proliferation and macrophage infiltration, immunofluorescence staining for Ki67, M1 and M2 markers was performed. Ki67 staining showed more proliferating cells when exosomes were applied to the wound area (PBS vs. DFATs-Exos/GelMA group: 3.20 ± 1.01% vs. 22.91 ± 2.47%). Exosomes loaded in GelMA further promote the cell proliferation (*P* < 0.05) (Fig. 7D). Co-staining of iNOS for M1 macrophages and CD206 for M2 macrophages on wound sections at days 7 showed a progressive transformation from M1 to M2. The DFATs-Exos/GelMA group exhibited fewer iNOS + M1 macrophages and more CD206 + M2 macrophages, suggesting a phenotype shift from M1 to M2(Fig. 8A and B). Western blot analysis of CD206 showed results consistent with immunofluorescence. DFAT-derived exosomes loaded into GelMA hydrogel were injected into diabetic wound sites. After 7 days, increased infiltration of CD206-positive M2 macrophages was observed compared to the blank control group. (Figure 8C and D) Full-length blots are presented in Supplemental Digital Material 3.

### DFATs-Exos promoted wound healing through Wnt/β-catenin pathway

Western blot significantly revealed activation of Wnt/β-catenin signaling when DFATs-Exos were added. (*P* < 0.05) Using GelMA hydrogel for the sustained release of DFATs-Exos, the DFATs-Exos/GelMA group exhibited faster wound healing with maximal Wnt/β-catenin pathway activation. (Figure 8E and G) Full-length blots are presented in Supplementary Fig. 4.

## Discussion

Diabetic wounds, particularly diabetic foot ulcers (DFUs), result from a complex interplay of factors, including chronic hyperglycemia, impaired angiogenesis, neuropathy, and immune dysfunction [18]. Persistent high glucose levels lead to vascular complications, reducing blood supply to the extremities and impairing wound healing. Additionally, a pro-inflammatory environment and oxidative stress contribute to tissue damage and delayed re-epithelialization. The resulting chronic wounds often exhibit impaired cell proliferation, reduced collagen deposition, and compromised angiogenesis. Addressing these pathological mechanisms remains a significant challenge for clinicians [19, 20]. In this context, our study explores a novel approach using DFATs-Exos encapsulated in GelMA hydrogel to enhance diabetic wound healing.

DFATs, derived from mature adipocytes through a ceiling culture process, represent a unique cell source with significant regenerative potential. Compared to other widely used seed cells, DFATs have significant advantages but are underexplored in the field of regenerative medicine. First, DFATs are derived harvested from adipose tissue obtained through via liposuction, providing a making them more economically and conveniently accessible cell source than compared to bone marrow or umbilical cord stem cells [21]. In contrast, ADSCs, despite being widely studied, constitute a minor cell population (less than 3%) in of adipose tissue and require extensive in vitro expansion of stromal vascular fractions (SVFs) to obtain sufficient exosome yields [22]. This process often results in cellular aging and the secretion of senescence-associated secretory phenotype (SASP) factors [23, 24]. DFATs, however, are harvested from mature adipocytes through the ceiling culture method, which allows for efficient recovery of primary cells from typical liposuction volumes. In this study, for instance, 120 ml of adipose tissue yielded 2 × 10⁸ DFATs and 400 µg of exosomes through ultracentrifugation. Furthermore, DFATs demonstrate robust differentiation potential toward endothelial and adipogenic lineages and secrete critical cytokines, including VEGF and HGF, with potent paracrine effects in vitro and in vivo [25]. Previous studies showed that fat grafts enriched with DFATs exhibit superior survival and vascularization compared to those supported by ADSCs, highlighting their promise as a seed cell in cell-assisted lipotransfer (CAL) [26]. However, clinical application of exogenous stem cells faces legal and ethical restrictions. Compared to cell transplantation, exosome-mediated cell-free therapy offers greater stability and long-term storability, lacks risk of tumor formation, and has lower immunogenicity [27, 28].

DFATs-Exos are extracellular vehicles with a bilayer lipid membrane structure, ranging in size from 30 to 150 nm, and containing a diverse array of bioactive molecules such as DNA, RNA, microRNA (miRNA), and proteins, which effectively regulate both long-distance and short-distance intercellular communication [29, 30]. In the field of wound healing, exosomes exhibit multiple beneficial functions, including inhibition of oxidative stress, suppression of inflammatory responses, promotion of angiogenesis, acceleration of epithelial regeneration, facilitation of collagen remodeling, and reduction of scar formation [31, 32]. However, the rapid degradation and clearance of exosomes after local application present a significant challenge to maintaining their biological activity, necessitating repeated administrations and complicating their therapeutic use [33]. To overcome this these limitations, we encapsulated DFATs-Exos in a GelMA hydrogel, a biomaterial recognized for its excellent biocompatibility, tunable degradation rates, and structural support capabilities. GelMA hydrogels not only facilitate the sustained release of encapsulated exosomes but also provide a moist wound environment that enhances cellular interactions and tissue repair [34]. Additionally, GelMA hydrogels act as a protective barrier, preventing direct contact between the wound and external contaminants, while forming stable porous structures to support cell growth and migration [35, 36, 37, 38]. In this study, a 10% porous GelMA hydrogel (pore size: 100–200 μm) was selected as the optimal carrier, as it effectively encapsulated exosomes while allowing sufficient space for cell migration and proliferation. This material’s ability to quickly transition to a gel state under 405 nm blue light also makes it suitable for treating irregular wound areas. In vitro experiments demonstrated that GelMA exhibited a stable porous structure, controlled degradation rates, and sustained exosome release, further highlighting its potential as a delivery system for exosome-based therapies in wound healing.

Our study provides compelling evidence that DFAT-Exos promote diabetic wound healing by activating the Wnt/β-catenin signaling pathway and enhancing key cellular and tissue-level processes. Characterization of DFATs and their exosomes confirmed their high purity and biological activity, as evidenced by their typical morphology, expression of stem cell markers, and enrichment of exosomal markers such as CD63, CD9, and TSG101. These findings underscore the therapeutic potential of DFAT-Exos as a cell-free treatment modality. The Wnt/β-catenin signaling pathway is a key regulator of cell proliferation, differentiation, and the cell cycle, and it plays a pivotal role in tissue regeneration and wound healing by promoting angiogenesis and epithelialization [39, 40, 41]. In this study, we observed that a high-glucose environment, which mimics diabetic conditions, led to significant inactivation of the Wnt/β-catenin signaling pathway, contributing to impaired cellular functions such as reduced proliferation and migration. However, treatment with DFAT-derived exosomes effectively restored Wnt/β-catenin signaling activity. Notably, the application of XAV939, a selective inhibitor of Wnt/β-catenin signaling, abrogated the pro-proliferative effects of DFAT-Exos on fibroblasts, confirming the pathway’s essential role in mediating these effects.

The structural and functional integration of DFAT-Exos with the GelMA hydrogel amplified their therapeutic effects. The 10% GelMA hydrogel not only provided a sustained-release platform for DFAT-Exos but also facilitated cellular uptake, as demonstrated by increased PKH26-labeled exosome internalization by fibroblasts over 24 h. This sustained delivery supported angiogenesis, evidenced by increased tube formation in HUVECs and elevated VEGF and HGF secretion, which are crucial for vascular remodeling in diabetic wounds. Moreover, histological analyses revealed enhanced re-epithelialization, collagen deposition, and granulation tissue formation in the DFAT-Exos/GelMA group, accompanied by a marked shift in macrophage polarization from the pro-inflammatory M1 phenotype to the anti-inflammatory M2 phenotype.

These findings highlight the synergy between DFAT-Exos and GelMA hydrogel in promoting wound repair through multifaceted mechanisms. Activation of the Wnt/β-catenin pathway not only rescued cellular functions impaired by hyperglycemia but also contributed to tissue regeneration by enhancing angiogenesis, reducing inflammation, and accelerating fibroblast differentiation into myofibroblasts. This integrated approach addresses the challenges of rapid exosome degradation and clearance, offering a sustained and potent therapeutic strategy for chronic diabetic wounds. Future studies should investigate the specific exosomal components responsible for pathway activation and explore their broader implications in other tissue repair contexts.

While this study demonstrates the therapeutic potential of DFAT-Exos encapsulated in GelMA hydrogel for diabetic wound healing, several limitations should be acknowledged. First, the specific bioactive components within DFAT-Exos responsible for activating the Wnt/β-catenin signaling pathway and promoting wound healing were not identified. Future studies should focus on isolating and characterizing these components, such as specific proteins, RNAs, or microRNAs, to elucidate their roles in tissue regeneration. Second, an intriguing observation from this study was the presence of perilipin-positive adipocytes in the dermis of the wounds 7 days after DFAT-Exos application, suggesting that DFAT-Exos may promote dermal adipogenesis (Supplemental Digital Material 5). This unique effect, not commonly reported with other mesenchymal stem cell-derived exosomes, highlights a potential advantage of DFAT-Exos in wound healing by restoring dermal fat layers, which play a critical role in skin integrity and repair. However, the mechanisms driving this phenomenon remain unclear and require further investigation to determine whether it is mediated by specific signaling pathways or cellular interactions. Finally, this study only explored three concentration gradients of DFAT-Exos, and the optimal dosage for wound healing remains undetermined. Further research is needed to systematically evaluate a wider range of exosome concentrations and their dose-response relationships, which could help optimize therapeutic strategies.

## Conclusion

This study demonstrated that DFAT-Exos encapsulated in GelMA hydrogel effectively promoted diabetic wound healing by enhancing angiogenesis, fibroblast activity, and macrophage polarization through activation of the Wnt/β-catenin signaling pathway. These findings highlight DFAT-Exos/GelMA as a promising cell-free therapy for diabetic wounds, warranting further research to optimize clinical applications.

## Electronic supplementary material

Below is the link to the electronic supplementary material.

Supplementary Digital Material 1: Uncropped full-length gels and blot of Fig. 2F and Fig.5D

Supplementary Digital Material 2: CCK-8 results of fibroblasts co-cultured with 10% GelMA

Supplementary Digital Material 3: Uncropped full-length gels and blot of Fig.5C, Fig. 8C

Supplementary Digital Material 4: Uncropped full-length gels and blot of Fig.4J, Fig. 8E

Supplementary Digital Material 5: Perilipin staining of wounds tissue slides

## Data Availability

The datasets used and/or analyzed during the current study are available from the corresponding author on reasonable request. All additional files are included in the manuscript.

## Abbreviations

DFATs-Exos: Dedifferentiated Fat Cells-derived Exosomes
ECs: Endothelial Cells
DFU: Diabetic Foot Ulcer
BMSCs: Bone marrow mesenchymal stem cells
HDFs: Human Dermal Fibroblasts
HG: High Glucose
ADSCs: Adipose-derived Stem Cells
