# Supplementary material for: Dedifferentiated fat cells-derived exosomes (DFATs-Exos) loaded in GelMA accelerated diabetic wound healing through Wnt/β-catenin pathway
Source: Stem Cell Res Ther. 2025 Feb 28;16:103. doi: 10.1186/s13287-025-04205-9 (PMC11871660; doi:10.1186/s13287-025-04205-9)

Supplementary Digital Material 1

Uncropped full-length gels and blot of Figure 2F.

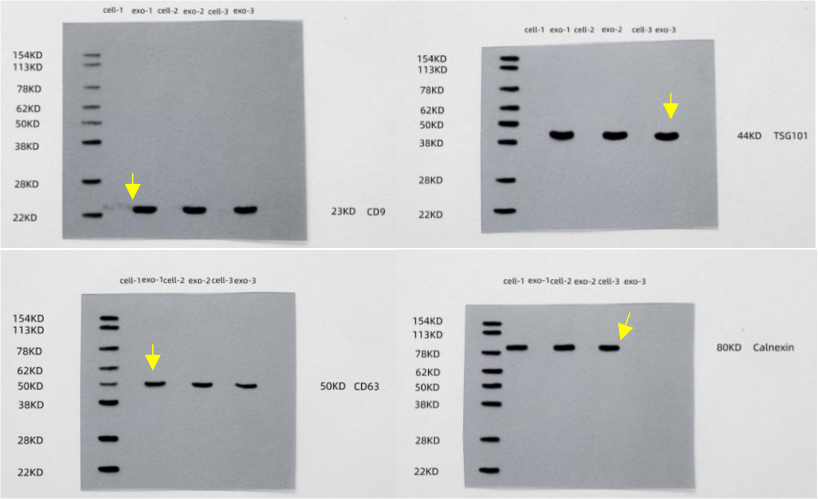

Uncropped full-length gels and blot of Figure 5D.

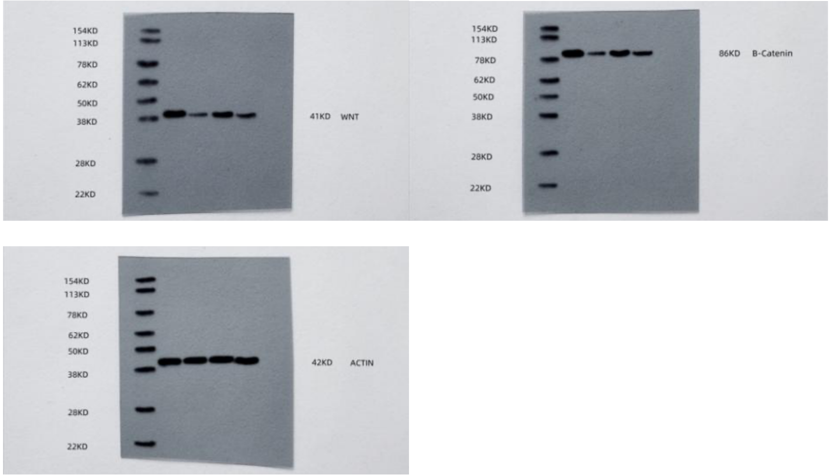

Supplement: Supplementary file 1 — Supplementary Digital Material 1: Uncropped full-length gels and blot of Fig. 2F and Fig.5D [file 13287_2025_4205_MOESM1_ESM.pdf]
