# Supplementary material for: Dedifferentiated fat cells-derived exosomes (DFATs-Exos) loaded in GelMA accelerated diabetic wound healing through Wnt/β-catenin pathway
Source: Stem Cell Res Ther. 2025 Feb 28;16:103. doi: 10.1186/s13287-025-04205-9 (PMC11871660; doi:10.1186/s13287-025-04205-9)
Supplement: Supplementary file 2 — Supplementary Digital Material 2: CCK-8 results of fibroblasts co-cultured with 10% GelMA [file 13287_2025_4205_MOESM2_ESM.pdf]

Supplementary Digital Material 2

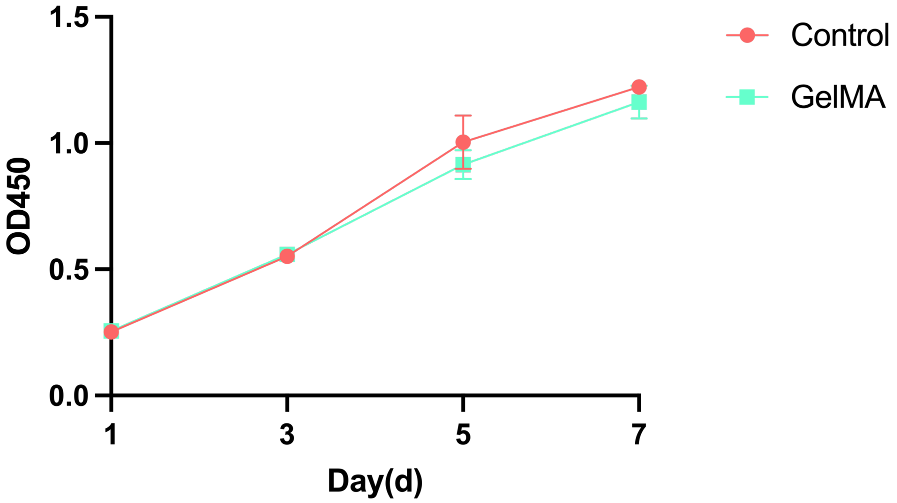

CCK-8 results of fibroblasts co-cultured with 10% GelMA. The 10% GelMA solution pose no cytotoxic effects on fibroblasts.
