# Supplementary material for: Dedifferentiated fat cells-derived exosomes (DFATs-Exos) loaded in GelMA accelerated diabetic wound healing through Wnt/β-catenin pathway
Source: Stem Cell Res Ther. 2025 Feb 28;16:103. doi: 10.1186/s13287-025-04205-9 (PMC11871660; doi:10.1186/s13287-025-04205-9)

Supplementary Digital Material 3
Uncropped full-length gels and blot of Figure 4J.

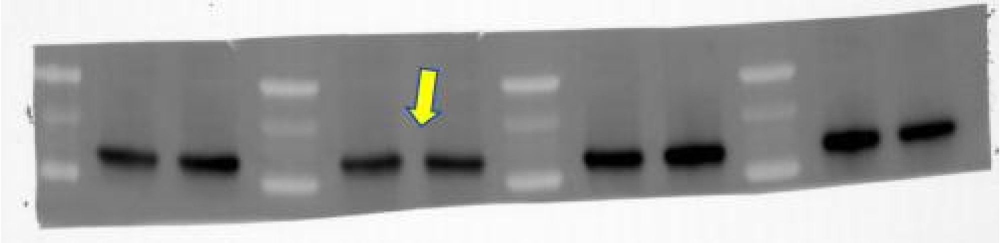

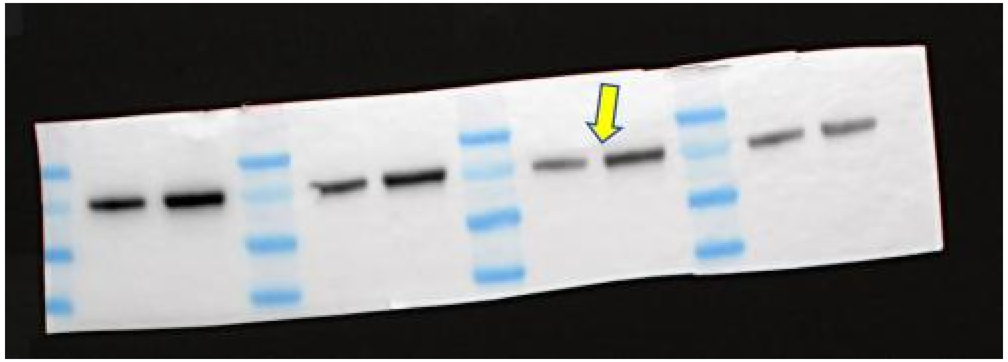

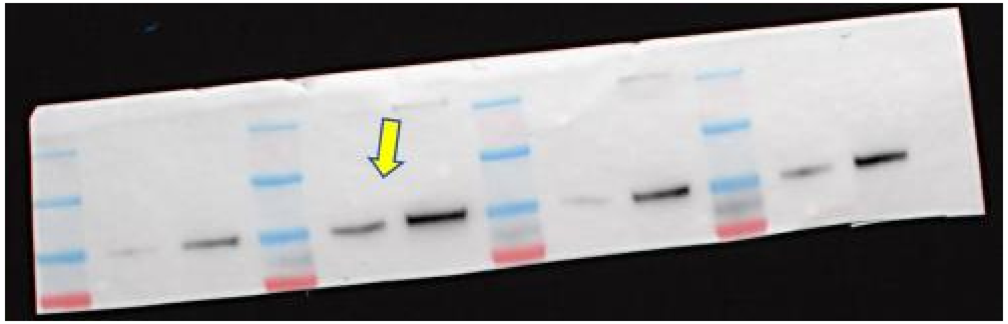

Uncropped full-length gels and blot of Figure 8E.

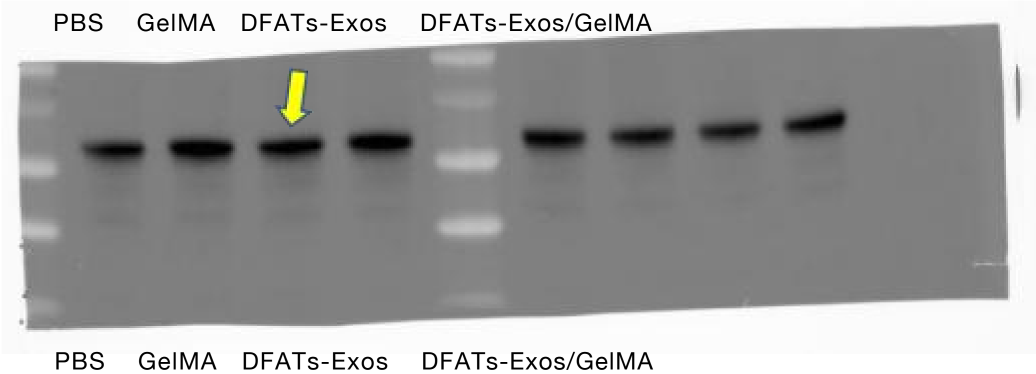

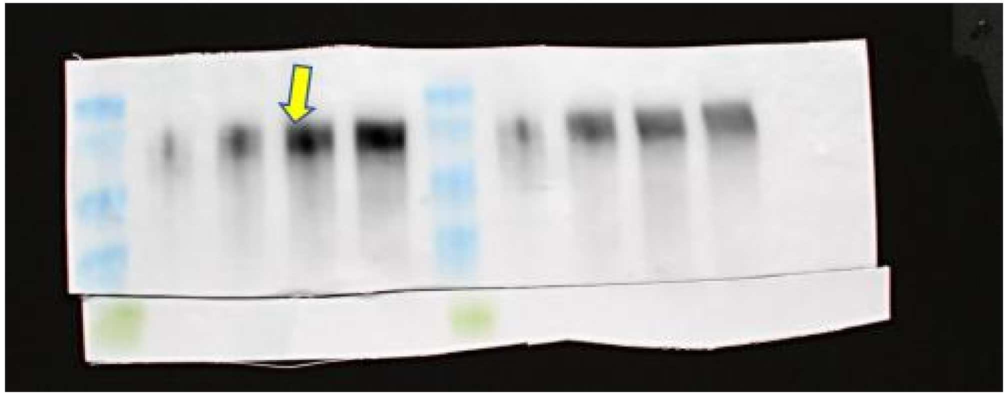

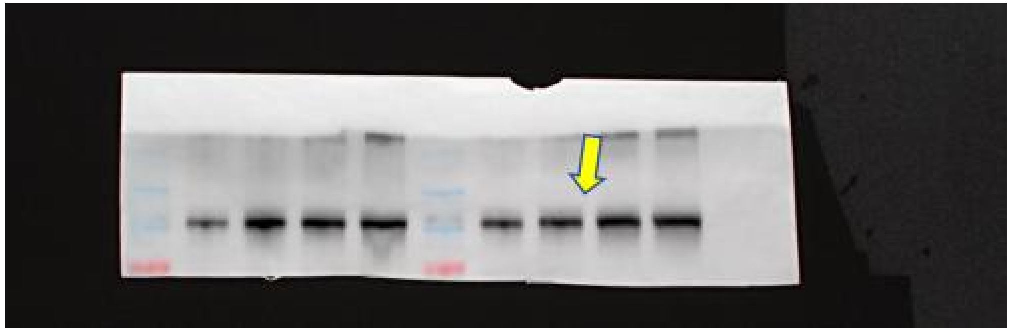

Supplement: Supplementary file 3 — Supplementary Digital Material 3: Uncropped full-length gels and blot of Fig.5C, Fig. 8C [file 13287_2025_4205_MOESM3_ESM.pdf]
