# Supplementary material for: Dedifferentiated fat cells-derived exosomes (DFATs-Exos) loaded in GelMA accelerated diabetic wound healing through Wnt/β-catenin pathway
Source: Stem Cell Res Ther. 2025 Feb 28;16:103. doi: 10.1186/s13287-025-04205-9 (PMC11871660; doi:10.1186/s13287-025-04205-9)

Supplementary Digital Material 4
Uncropped full-length gels and blot of Figure 5A.

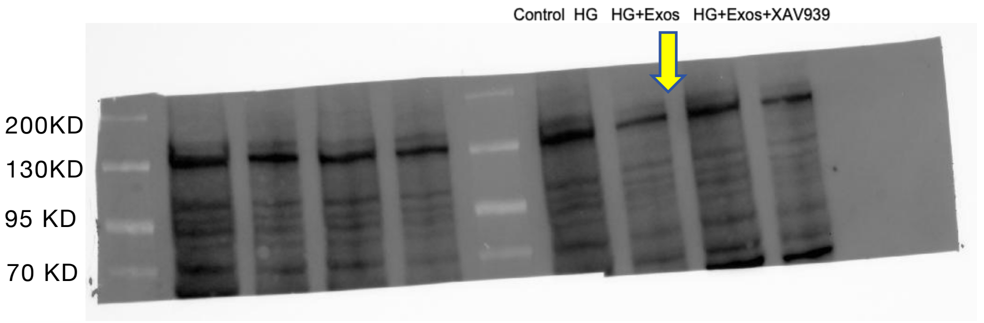

Col 1 144KD

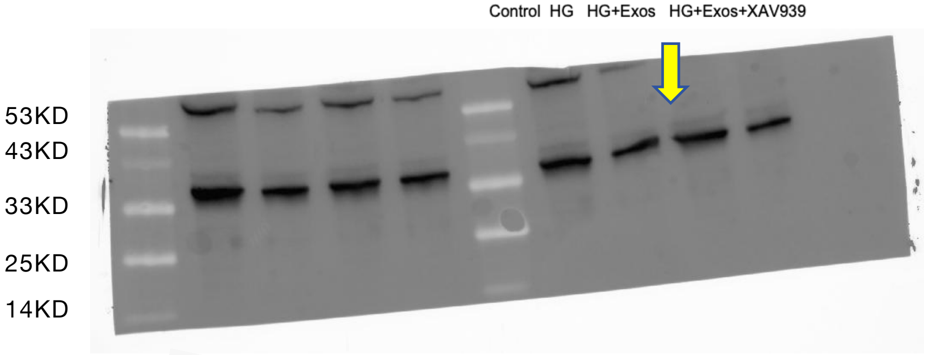

α-SMA 42KD

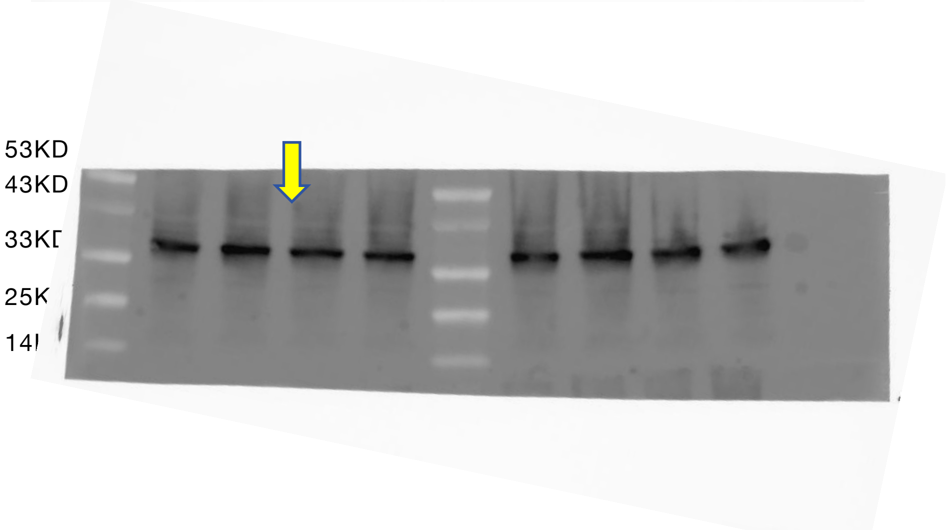

GAPDH 36KD

Uncropped full-length gels and blot of Figure 8C.

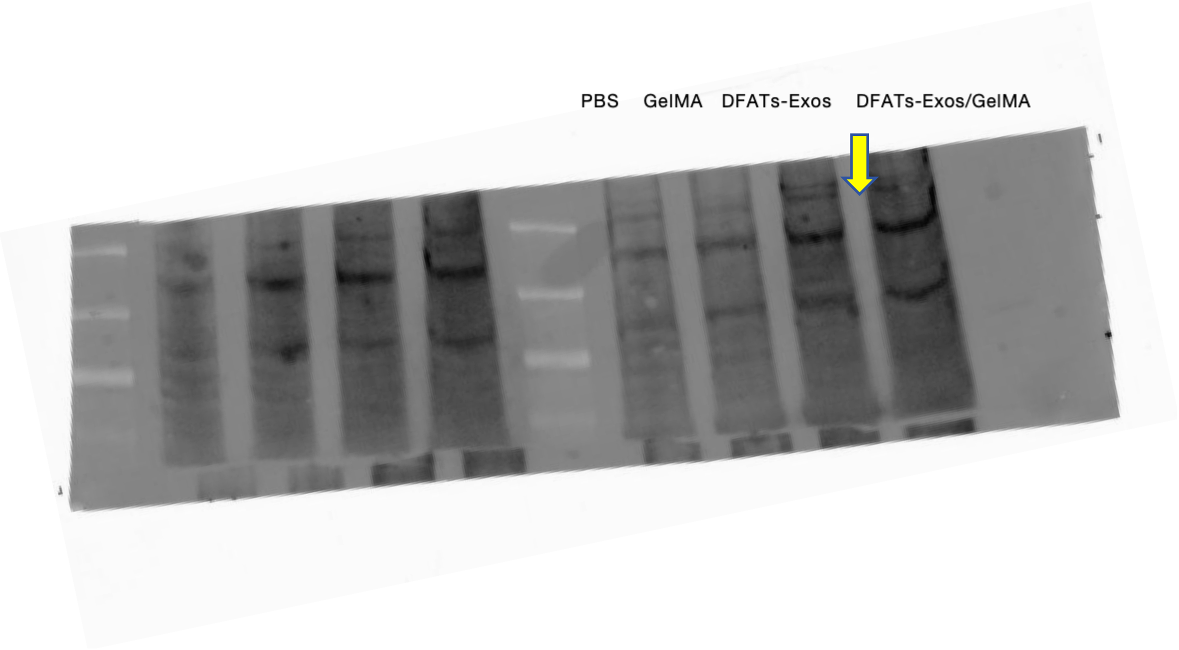

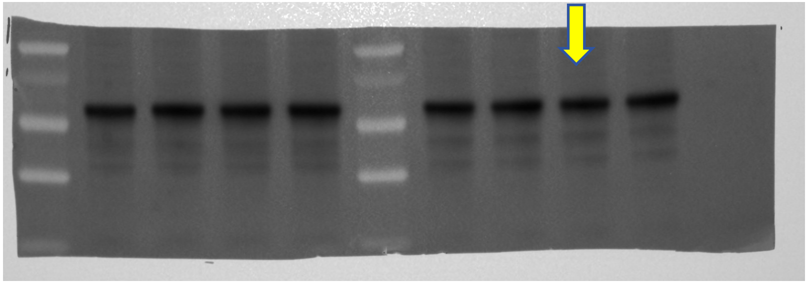

Supplement: Supplementary file 4 — Supplementary Digital Material 4: Uncropped full-length gels and blot of Fig.4J, Fig. 8E [file 13287_2025_4205_MOESM4_ESM.pdf]
