# Supplementary material for: Dedifferentiated fat cells-derived exosomes (DFATs-Exos) loaded in GelMA accelerated diabetic wound healing through Wnt/β-catenin pathway
Source: Stem Cell Res Ther. 2025 Feb 28;16:103. doi: 10.1186/s13287-025-04205-9 (PMC11871660; doi:10.1186/s13287-025-04205-9)

Supplementary Digital Material 4

A: Perilipin staining for DFATs-Exos/GelMA group.

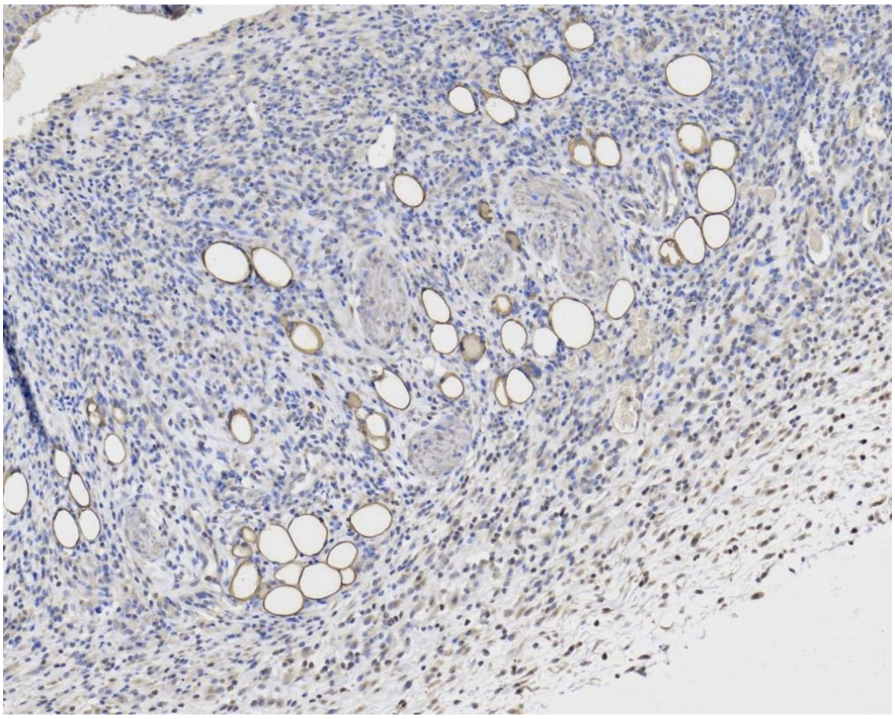

B: Perilipin staining for DFATs-Exos group.

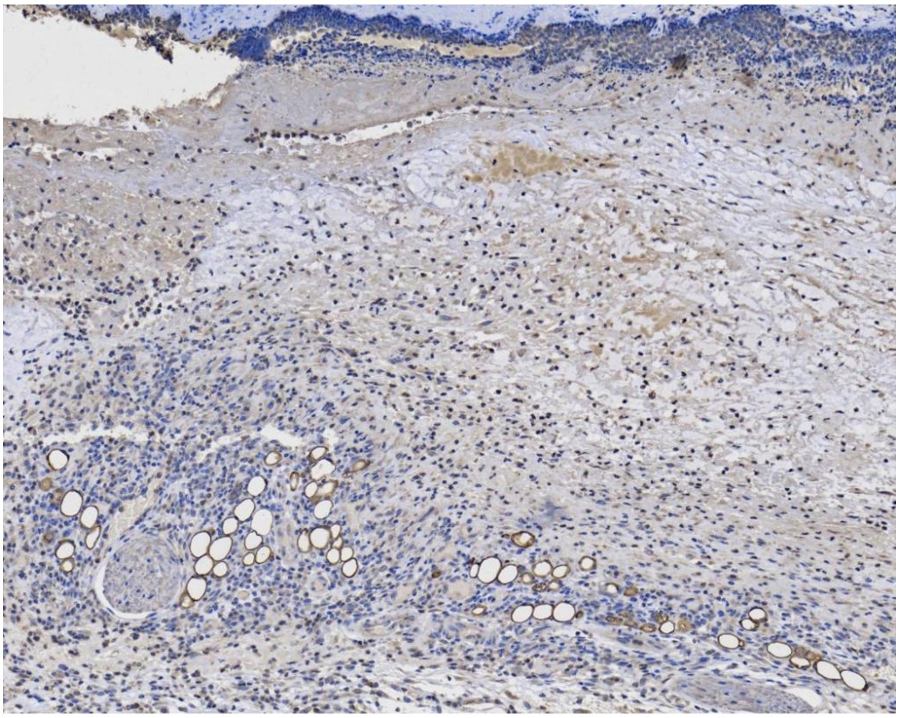

C: Perilipin staining for GelMA group.

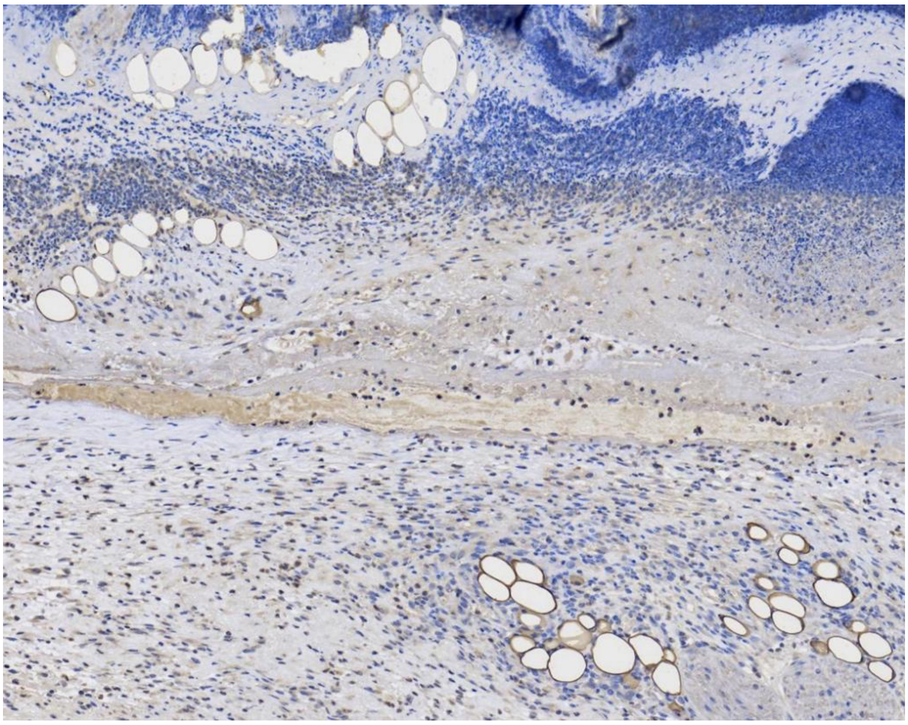

D: Perilipin staining for PBS group.

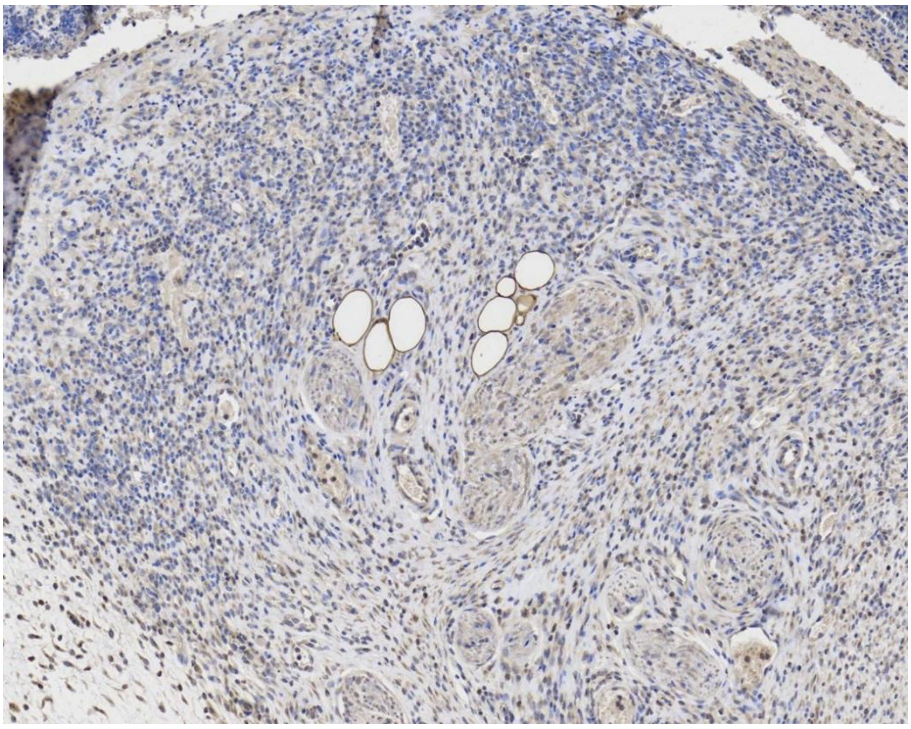

Supplement: Supplementary file 5 — Supplementary Digital Material 5: Perilipin staining of wounds tissue slides [file 13287_2025_4205_MOESM5_ESM.pdf]
